# *BmMBNL* Knockout Disrupts Molting of Silkworm, *Bombyx mori*, Through Inhibiting Expressions of Chitin Synthetase and Chitinase

**DOI:** 10.3390/insects17080849

**Published:** 2026-08-14

**Authors:** Huan Dong, Zihan Meng, Mengyao He, Yujuan Zhou, Ding Tu, He Wang, Xian Li, Yiwen Liang, Wenjuan Fan, Chaopin Zhu, Qingyou Xia, Feng Wang

**Affiliations:** Integrative Science Center of Germplasm Creation in Western China (CHONGQING) Science City, Biological Science Research Center, Southwest University, Chongqing 400715, China

**Keywords:** *Bombyx mori*, *BmMBNL*, CRISPR/Cas9, molting, chitinase, chitin synthase

## Abstract

Insects periodically undergo molting to replace their old epidermis for growth and morphological adaptation. Muscleblind-like (MBNL) is a multifunctional protein involved in miRNA processing. To explore the role of miRNA processing in insect molting, we characterized BmMBNL in *Bombyx mori*. *BmMBNL* exhibited spatiotemporal expression across tissues and developmental stages, with the highest levels in the epidermis and upregulation during molting. CRISPR/Cas9-mediated knockout of *BmMBNL* (*BmMBNL*-KO) caused molting defects at the 3rd and 4th instar larvae, resulting in reduced size and eventual lethality. Moreover, *BmMBNL*-KO individuals showed a thinner chitin layer between the new and old epidermises. RT-PCR and transcriptome analyses revealed upregulation of miR-1, miR-71, miR-263, miR-8-5p, and miR-2a-3p, which suppressed chitinase expression, ultimately leading to molting failure. This study provides novel insights into the potential correlation between *BmMBNL*, miRNA abundance and insect molting in *Bombyx mori*.

## Simple Summary

Insects rely on molting to replace their old cuticle for growth, a process tightly regulated at the molecular level. However, the role of miRNA processing in this event remains poorly understood. In this study, we investigated the function of Muscleblind-like (MBNL) protein in the silkworm *Bombyx mori*, which is involved in miRNA biogenesis. We found that *BmMBNL* is highly expressed in the epidermis and upregulated during molting. CRISPR/Cas9 knockout of *BmMBNL* caused severe molting defects, reduced body size, and larval lethality, accompanied by decreased ecdysone titers and impaired chitin layer formation. Mechanistically, loss of *BmMBNL* led to the upregulation of several miRNAs (miR-1, -71, -263, -8-5p, -2a-3p), which suppressed chitinase expression and disrupted normal molting. These findings reveal a novel miRNA-mediated regulatory axis that controls insect molting. Our work not only advances fundamental understanding of insect development but also provides potential targets for pest management and strategies to improve sericulture productivity, thus offering both ecological and economic benefits to society.

## 1. Introduction

The insect exoskeleton, mainly constituted by the cuticle, supports multiple organs and fulfills diverse physiological functions. It is composed of an extracellular matrix secreted by epidermal cells and strengthened by substances like chitin and epidermal proteins. The cuticle reduces water loss, provides mechanical protection, and acts as a physical barrier against pathogenic bacteria. This outer cuticle is essential for insect growth, development, reproduction, flight, and other physiological functions. However, the limited extensibility of the cuticle may hinder larval growth. Insects undergo periodic molting to overcome this limitation, shedding their old cuticle and developing a new, larger, and more flexible one. Since the successful completion of the molting process is essential to the life cycle of silkworm, it is particularly important for their growth and development.

Muscleblind-like (MBNL) belongs to the muscleblind-like protein family and is a group of ancient metazoan RNA-binding proteins [1]. It is essential for a variety of reprogramming pathways during postnatal heart development in vertebrates [2], and it is also crucial for the differentiation of stem cells [3], red blood cells [4], and myofibroblasts [5]. The MBNL protein was initially identified as a regulator of alternative splicing, regulating the alternative splicing of multiple pre-mRNAs [6,7,8]. In addition to its splicing regulatory function, MBNL also influences gene expression by facilitating the transport of cellular mRNA [9,10,11], maintaining mRNA stability [12,13], and processing microRNAs (miRNAs) [14]. MBNL inhibits or activates splicing, depending on the binding location. When MBNL binds to an exonic splicing enhancer, it encourages the inclusion of certain exons, whereas binding to an exonic splicing silencer can suppress the splicing of specific exons. Within the cytoplasm, MBNL’s binding to the 3′ untranslated region (UTR) may assist in directing signal sequences to mRNAs in the rough endoplasmic reticulum or in targeting mRNAs to membrane-rich organelles for localized translation via actin, microtubule, or intermediate filament-based molecular motors, which may include synapses, neuromuscular junctions, or plasma membranes, depending on the cell type. MBNL is also involved in the subtype-specific localization of mRNA, where its binding sites in the distal 3′ UTR are essential for proper mRNA targeting.

Among mammals, the MBNL family consists of three members: MBNL1, MBNL2, and MBNL3 [15]. MBNL1 promotes muscle differentiation, whereas MBNL2 plays a part in the RNA-dependent protein localization mechanism. Unlike MBNL1, MBNL3 inhibits the expression of muscle differentiation markers. Dysfunction of the MBNL1 protein has been identified as a contributing factor to myotonic muscular dystrophy [16,17]. Abnormal amplification of CUG and CCUG at the RNA level plays a key role in DM1 and DM2 induced by repeated amplification of CTG and CCTG [18,19,20,21], whereas MBNL1 is sequestered by RNA containing amplified CUG/CCUG repeats, affecting the binding of MBNL1 to normal pre-miRNA targets, resulting in splicing defects. The MBNL protein has been found in nuclear foci, where it is sequestered by RNA carrying these amplified CUG or CCUG repeats [22,23].

Since the discovery of miRNA, a multitude of studies have highlighted its pivotal role in the regulation of diverse biological processes within living organisms. In particular, miRNA has been shown to exert a substantial regulatory effect on the growth and development of insects, especially during the stages of molting or metamorphosis. Dysregulation of miRNA can impact the synthesis and degradation of chitin during molting, consequently leading to molting defects in insects. The MBNL protein is an RNA-binding protein that regulates the alternative splicing of pre-mRNA, mediates the transport and stability of cellular mRNA, and processes microRNAs (miRNAs) to influence gene expression. Could MBNL influence the occurrence of molting defects in silkworms through the modulation of miRNA processing? To explore this possibility, we intend to employ CRISPR/Cas9 gene-editing technology to generate *MBNL* gene knockout mutants, obtain individuals with loss of *MBNL* function, and subsequently evaluate the role of MBNL in the regulation of molting in silkworms.

Lepidoptera, the second largest order in the class Insecta, plays a significant role in agriculture and forestry because over 60% of them are considered pests. *Bombyx mori*, which is recognized by the International Invertebrate Society as a representative model for Lepidoptera, is distinguished by the completion of its full genomic sequencing, making it a unique entity within this order. This accomplishment offers a valuable research platform for the study of functional genes related to Lepidopteran pests [24]. Exploring the intrinsic gene regulatory mechanism of insect molting behavior may facilitate the discovery of new insecticide targets and the development of innovative insecticides.

Here, we propose a hypothesis that *BmMBNL* may serve as an upstream factor associated with silkworm molting via modulating miRNA abundance and subsequent chitin metabolism in epidermal tissue. Although the linkage between miRNAs and chitin metabolism during insect molting has been well documented, the upstream molecular factors governing this regulatory cascade remain poorly characterized. This study aims to identify and functionally characterize *BmMBNL* in silkworm molting.

## 2. Materials and Methods

### 2.1. Bioinformatics Analysis

Full-length cDNA and protein sequences of *BmMBNL* were obtained from Silkbase (http://silkbase.ab.a.u-tokyo.ac.jp/, accessed on 8 January 2022). Characterization and sequence analyses of *BmMBNL* were performed using the ExPASy server (http://www.expasy.org/, accessed on 9 March 2022), SMART analysis service (http://smart.embl-heidelberg.de/, accessed on 9 March 2022), and ClustalX version 1.8.

### 2.2. Silkworm Rearing and Materials Collection

The silkworms were reared at the Integrative Science Center of Germplasm Creation Western China (CHONGQING). They were fed fresh mulberry leaves under a 12 h light/12 h dark photoperiod at 25 ± 1 °C and 70 ± 5% humidity. Tissue dissection was performed on ice, and the dissected tissues were immediately frozen in liquid nitrogen. Tissue samples, including the head, epidermis, midgut, silk gland, fat body, malpighian tubules, testes, and ovaries, were dissected from fifth-instar larvae on day 3. Whole-body samples of larvae at different developmental stages were harvested to generate the temporal expression profile. All samples were stored at −80 °C until further analysis.

### 2.3. RT-qPCR

RNA was extracted using TRIzol reagent (Invitrogen, Waltham, MA, USA) and then stored at −80 °C. cDNA samples were synthesized using M-MLV reverse transcriptase (Takara, Maebashi, Japan) at 42 °C and stored at −80 °C. The cDNA samples were normalized by using *Bombyx mori sw22934* as an internal control. This gene exhibits stable expression across multiple tissues and developmental stages of *Bombyx mori*, and has been widely employed for RT-qPCR analysis in silkworm studies [25]. RT-qPCR was performed with SYBR Premix Ex Taq (Takara, Maebashi, Japan) on a CFX96 Touch real-time PCR detection system (Bio-Rad, Hercules, CA, USA). Each 20 μL reaction contained 100 ng cDNA and 0.8 μM primers. Thermal cycling conditions were as follows: 95 °C for 3 min (initial denaturation); 40 cycles of 95 °C for 5 s, 60 °C for 30 s. The RT-qPCR primers used are listed in Table 1. Three independent replicates were used for each RT-qPCR.

### 2.4. Vector Construction

The exonic sequence of *BmMBNL* was selected as the target region of the knockout, and the targeted sequence was entered into the website (https://crispr.cos.uniheidelberg.de/index.html, accessed on 16 May 2022), which predicted three potential knockout target sites. The sgRNA was ligated to the piggyBac [3×P3-EGFP-SV40-TTTTTT-U6] base vector [26], and the ligation product was transfected into competent cells, cultured overnight, and screened for positive clones. The screened positive bacterial liquid was sent for sequencing. BioEdit software (v7.2.5) was used to align the sequencing results with the full-length sequence of *BmMBNL*, and the positive clones with knock-out effect were selected for plasmid extraction, for which the final concentration should be more than 500 ng/μL. The plasmid was injected into silkworm embryos within one week.

### 2.5. Epidermal Section Staining

Freshly dissected epidermis was fixed in 4% paraformaldehyde (Biosharp, Beijing, China) for 24 h. After 1×PBS washing, tissues underwent gradient ethanol dehydration. Samples were cleared with an ethanol–xylene mixture and pure xylene (Sangon Biotech, Shanghai, China), followed by paraffin (Sangon Biotech, Shanghai, China) infiltration at 65 °C, embedding, and sectioning at 7 μm. Sections were stretched at 37 °C, mounted on adhesive slides, and baked at 60 °C for 30 min. All slides were sequentially dewaxed. Deparaffinized sections were stained using an H&E staining kit (Beyotime, Shanghai, China). Sections were differentiated in 1% acid alcohol, counterstained, dehydrated, cleared in xylene, mounted with neutral balsam, and observed under a light microscope (Olympus, Tokyo Japan). Deparaffinized sections were incubated with Fluorescent Brightener 28 (Acmec, Shanghai, China) in the dark for 10 min. After three washes with 1×PBS on a shaker in the dark, sections were mounted with anti-fade mounting medium and observed under fluorescence microscopy (Olympus, Tokyo, Japan).

### 2.6. Scanning Electron Microscopy

Freshly dissected epidermal samples were fixed in 4% paraformaldehyde for 24 h, rinsed three times with 1×PBS, and then subjected to freeze-drying. Cocoon morphology was assessed using a scanning electron microscope (JSM-5000; JEOL, Tokyo, Japan). The cocoon samples of each group were glued to conductive carbon tape and coated with a thin layer of platinum (NeoCoater MP-19020NCTR; JEOL). SEM observations were performed at 25 ± 1 °C.

### 2.7. Transcriptome Data Analysis

Library construction and high-throughput sequencing of miRNA and RNA-seq libraries were performed by Qiantang Biotechnology (Suzhou) Co., Ltd. (Suzhou, China). The samples used for sequencing were epidermal tissues collected from fourth-instar molting silkworm larvae. Raw RNA-seq data were processed with fastp (v0.23.2) to remove adapters and low-quality sequences. Clean reads were mapped to the *Bombyx mori* reference genome using STAR (v2.7.10a), and gene read counts were summarized by featureCounts (V2.0.3). Differential expression analysis was performed between *BmMBNL*-KO and control epidermal samples using DESeq2 (v1.38.3). Genes with |log2(fold change)| > 1 and p-adj < 0.05 were identified as differentially expressed genes (DEGs). Heatmaps of DEGs were generated with the ggplot2 package (v3.4.4). GO and KEGG enrichment analyses were carried out using clusterProfiler (v4.6.2), and significantly enriched terms were screened based on hypergeometric distribution tests (*p*-value < 0.05).

### 2.8. Statistics and Reproducibility

All statistical analyses were performed using GraphPad Prism 9.0, and the data are presented as the means ± SD. The mean cycle threshold (Ct) value was converted to the relative expression level using the 2^−ΔΔCt^ method [27,28]. Statistical analyses of the expression levels were performed using two-tailed unpaired Student’s *t*-test. Significant differences were defined as * *p* < 0.05, ** *p* < 0.01, *** *p* < 0.001, **** *p* < 0.0001.

## 3. Results

### 3.1. Expression Analysis of BmMBNL Genes in Silkworm

**Figure 1:**
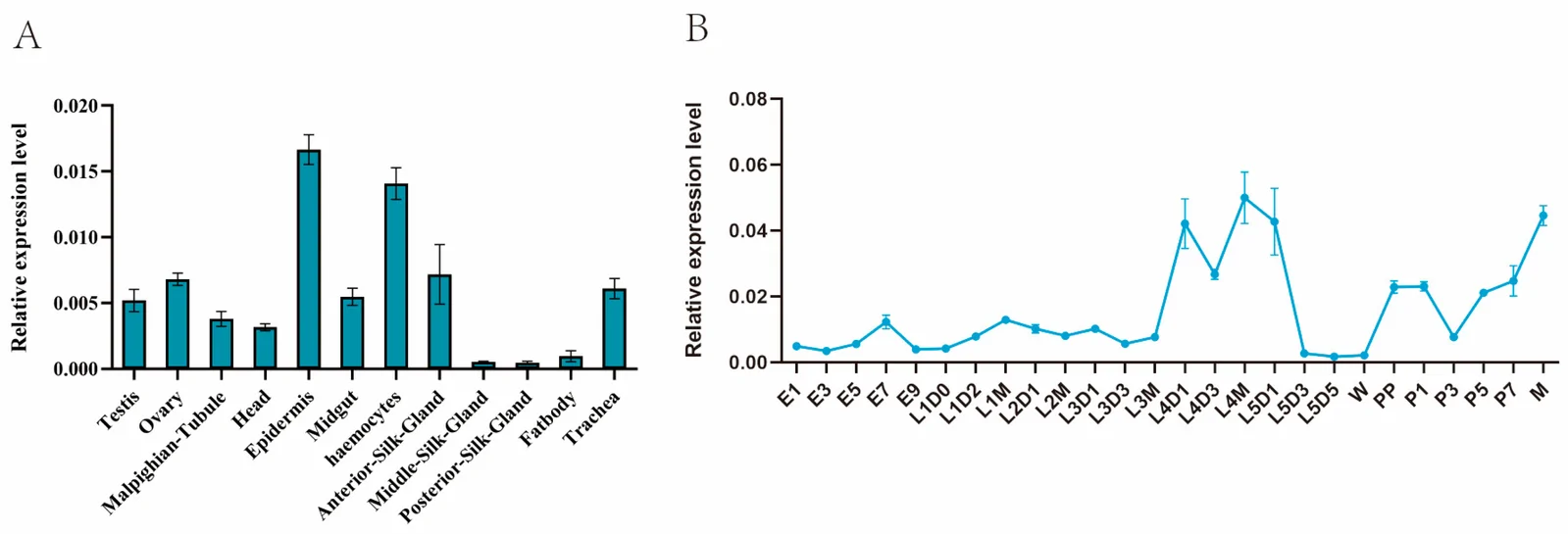
Spatial–temporal expression analysis of *BmMBNL*. (**A**): Expression pattern of *BmMBNL* in tissues of silkworm 5L3D. (**B**): Expression of *BmMBNL* at different stages of the silkworm primordium.

The MBNL protein is an RNA-binding protein that regulates the alternative splicing of pre-mRNA, mediates cellular mRNA transport and stability, and affects microRNA (miRNA) processing to influence gene expression. The function of the *BmMBNL* gene in *Bombyx mori* has not yet been reported. Through homologous alignment of the Drosophila *mbl* sequence, the homologous gene *MBNL* (*KWMTBOMO01862*) of *mbl* in *Bombyx mori* was obtained. To explore the function of the *BmMBNL* gene in silkworms, we performed RT-qPCR to analyze its expression levels in various tissues, including the head, epidermis, testis, ovary, midgut, fat body, malpighian tubules, silk gland, hemocytes, and trachea, on the third day of the fifth instar stage (Figure 1A). The results revealed that *BmMBNL* is expressed across multiple tissues, with the highest expression levels detected in the epidermis and the lowest in the silk gland. To further investigate the role of *BmMBNL* in the developmental process of silkworms, we analyzed the expression patterns of *BmMBNL* across each instar stage of the silkworm’s development. The results showed (Figure 1B) that the expression levels of *BmMBNL* fluctuate during the molting and feeding phases of each larval instar.

### 3.2. Construction of BmMBNL Knockout Mutants

**Figure 2:**
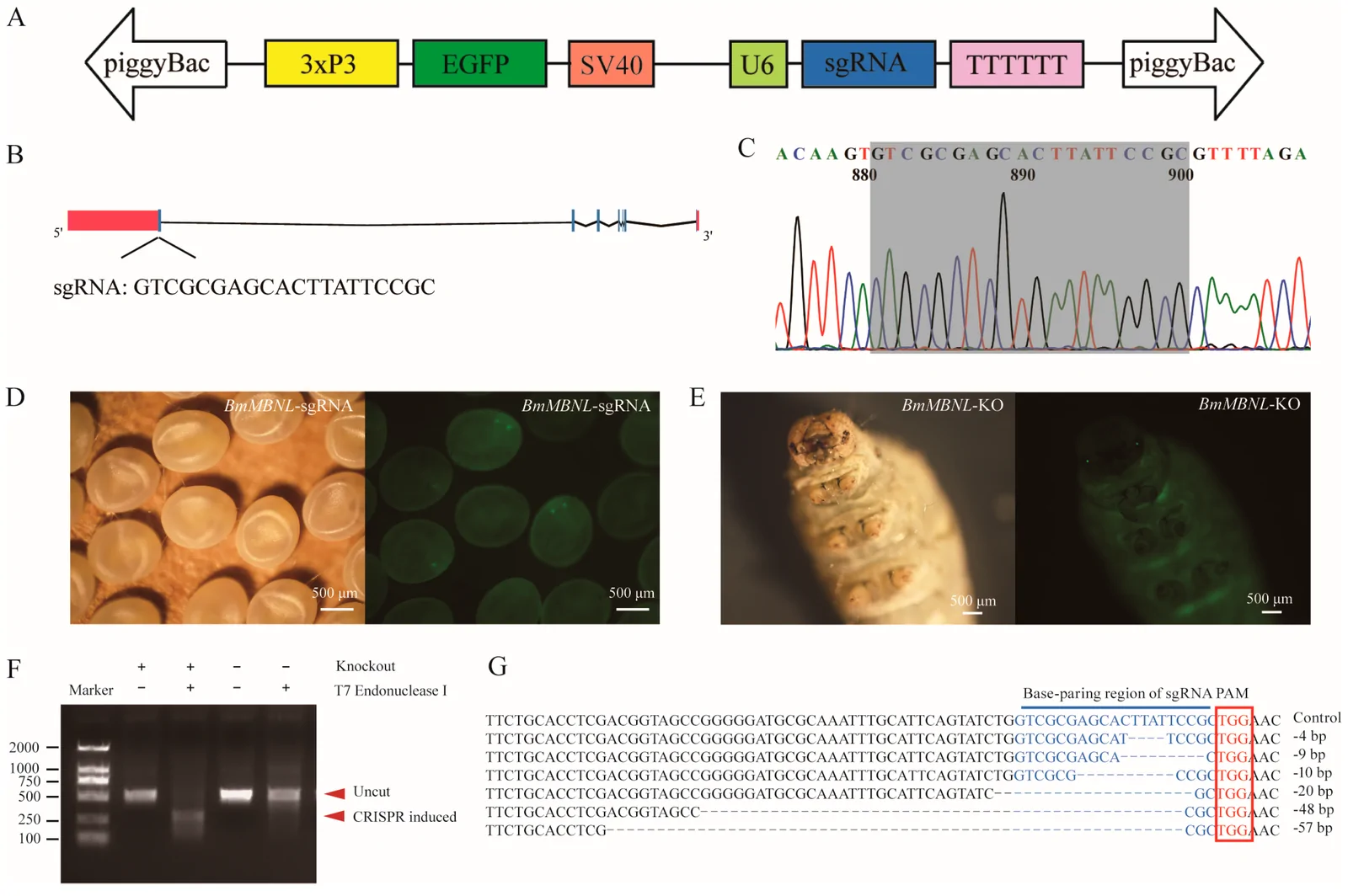
Construction of knockout strain. (**A**): Schematic diagram of knockout vectors. (**B**): Schematic diagram of *BmMBNL* knockout vector sgRNA design site. (**C**): Sequencing results of knockout vectors. (**D**): The positive embryos of *BmMBNL*-sgRNA. (**E**): The *BmMBNL* knockout positive individuals. (**F**): T7 endonuclease I (T7E1) verified the knockout status. The band at approximately 500 bp corresponds to the undigested parental PCR amplicon. Bands around 250 bp are cleavage fragments generated by T7E1 digestion of mismatched heteroduplex DNA, indicating deletion mutations at the target site. (**G**): Part of the mutant Sequences. The wild-type sequence is shown at the top. The number after the sequence indicates the number of bases of the mutation recovered by the sequence. The bases marked in the red box represent the PAM sequence. Deletions are indicated by dashed lines.

Based on the sequence analysis conducted on the CCTOP website, three sgRNAs were designed and ligated into a knockout vector (Figure 2A,B). Sequencing results confirmed the successful construction of the knockout vector (Figure 2C). This vector was then microinjected into silkworm eggs, and the G1 generation eggs were screened under a fluorescence microscope. The results showed that we successfully identified sgRNA-positive individuals with green fluorescent eyes (Figure 2D). The sgRNA-positive individuals were crossed with Cas9-overexpressing silkworm strains, and F1-generation individuals exhibiting green fluorescence in both eyes and body were selected as *BmMBNL* knockout transgenic silkworms (Figure 2E). Genomic DNA was extracted from *BmMBNL*-KO individuals, and genomic PCR and sequencing were used to verify the knockout of *BmMBNL*. The results revealed that the *BmMBNL*-KO genome exhibited deletions of varying lengths (Figure 2E), indicating that the *BmMBNL* gene had been successfully knocked out.

### 3.3. BmMBNL Deficiency Leads to Molting Defect of Silkworm

**Figure 3:**
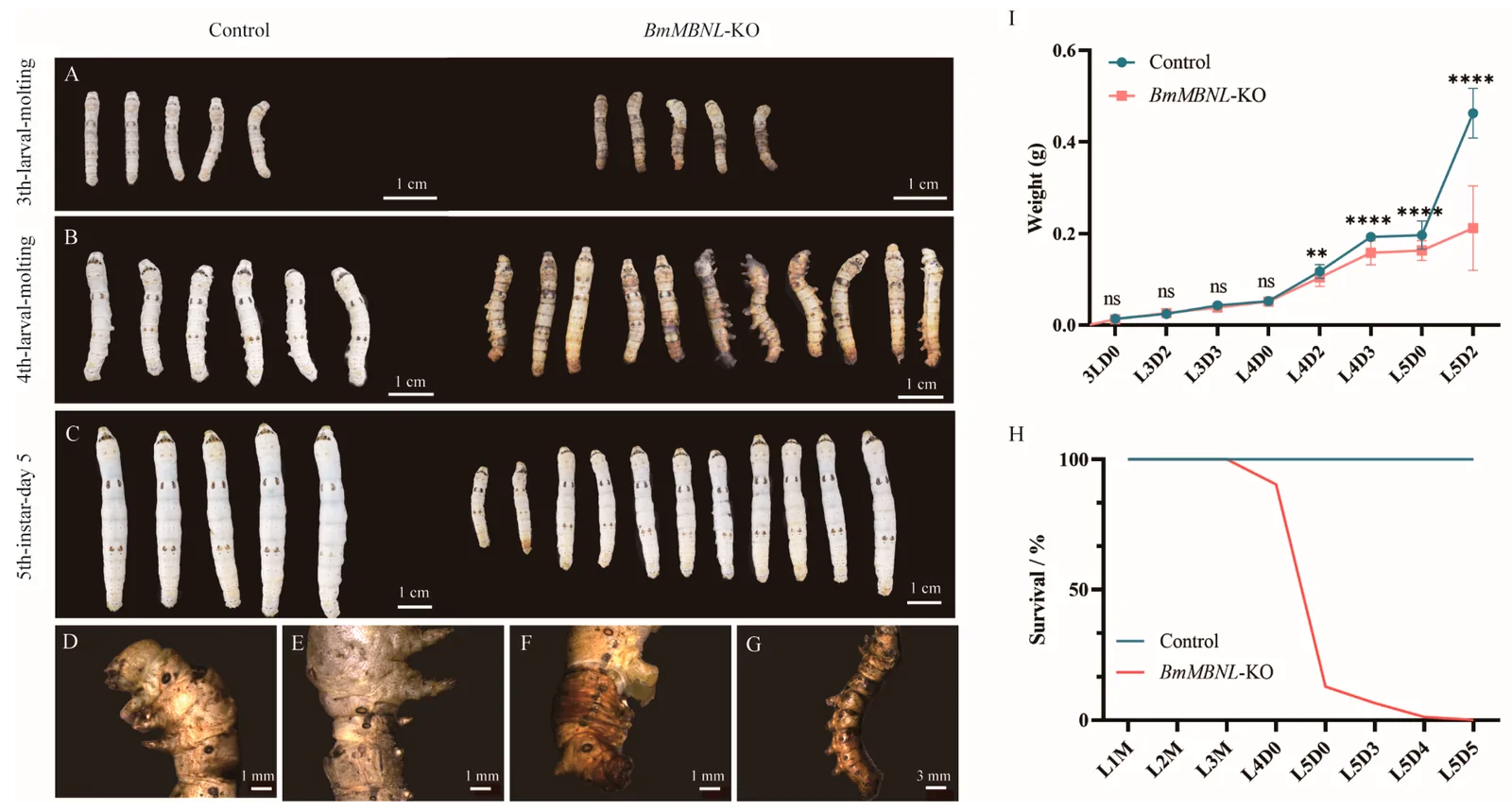
Phenotypic observation of *BmMBNL*-KO individuals. (**A**): Phenotype observation of the third molting. (**B**): Phenotype observation of the fourth molting. (**C**): Phenotype observation of 5th-instar-day-3. (**D**–**G**): Peripheral details of *BmMBNL*-KO. (**I**): Weight statistics of larvae (Data are shown as mean ± SD. Statistical analysis was conducted using Student’s *t*-test. ns, not significant; ** *p* < 0.01, **** *p* < 0.0001.). (**H**): Descriptive survival curves of *BmMBNL*-KO silkworm at different developmental stages.

Compared with control group silkworms, there was no significant difference in the hatching rate of *BmMBNL*-KO silkworms. During the rearing process, there were no significant differences in growth status, body shape, or weight between *BmMBNL*-KO silkworms and the control group before the third instar molting stage. However, during the molting period, *BmMBNL*-KO silkworms exhibited a molting disorder phenotype. Statistically, approximately 9.6% of the silkworms were unable to molt normally during the 3rd instar dormant stage (Figure 3A), leading to death; approximately 77.5% could not complete molting during the fourth instar molting period (Figure 3B); the remaining 12.7% were able to molt during the fourth instar molting period, but these individuals were smaller and died successively during the fifth instar feeding period (Figure 3C). Three main phenotypes were observed in *BmMBNL* knockout silkworms: non-molting (Figure 3G), semi-molting (Figure 3D–F), and smaller individuals (Figure 3C). Observation of a molting-disordered individual under a super-depth-of-field microscope revealed that the old head capsule had detached (Figure 3D), part of the body had shed its skin to the thorax or tail (Figure 3F), and constriction marks were visible. These results indicate that the deletion of *BmMBNL* in silkworms leads to molting disorders, ultimately causing the death of the silkworms. The body weight of *BmMBNL*-KO individuals was calculated throughout their growth process. As shown in Figure 3I, the body weight of *BmMBNL* knockout individuals was significantly lower than that of the control silkworms from the second day of the fourth instar until death. Statistical analysis of mortality at different stages revealed that a large number of *BmMBNL*-KO individuals died during the fourth molting stage, and all individuals had died by the fifth instar day 5 (Figure 3H).

**Figure 4:**
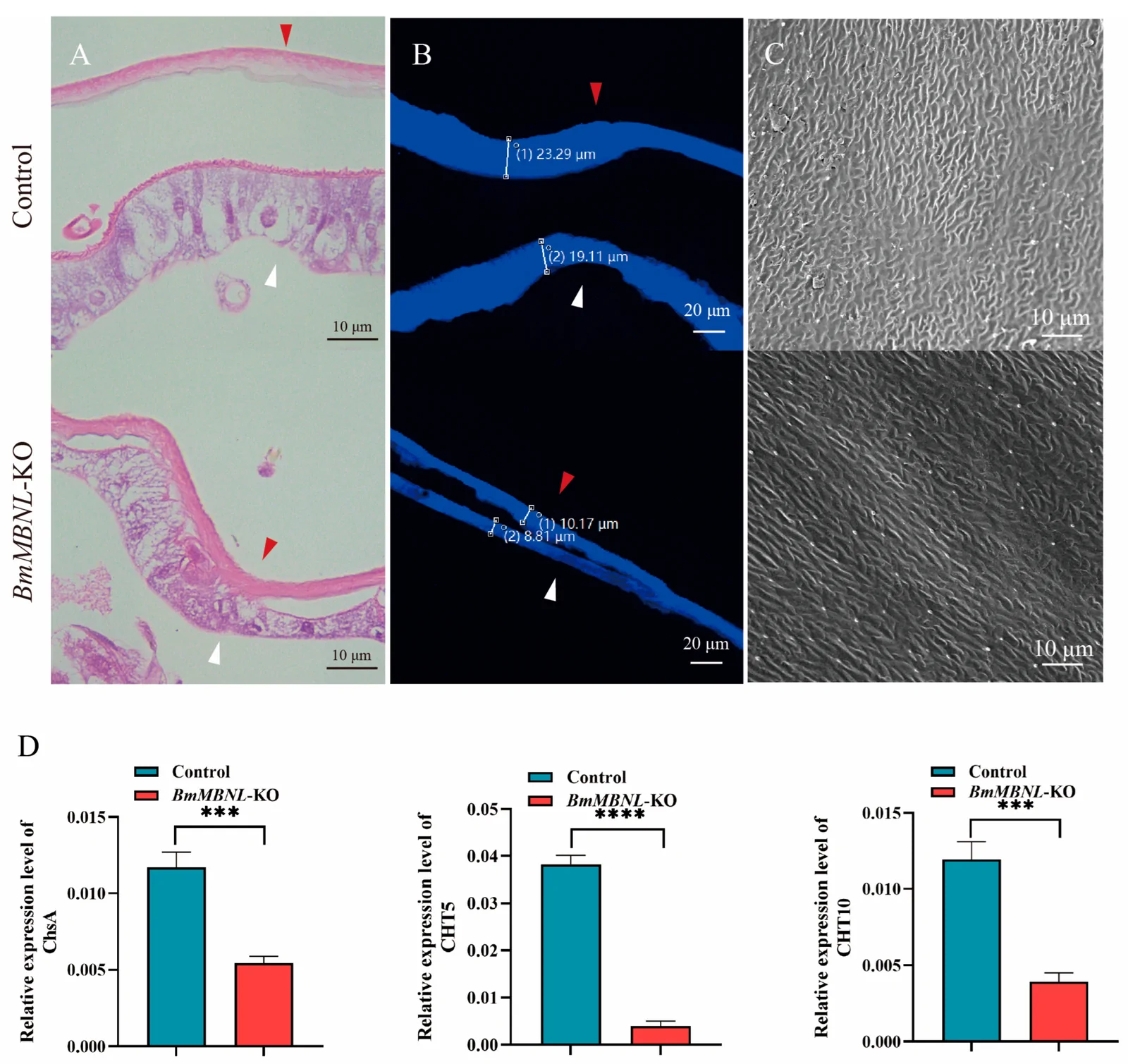
Histological staining, scanning electron microscopy and RT-qPCR quantification of chitinase-related genes in epidermal tissues from *BmMBNL-KO*. (**A**): Hematoxylin and eosin (H&E) staining; red arrow refers to the old epidermis, white arrow refers to the new epidermis; purple indicates epidermal cells, and pink indicates the old cuticle. (**B**): Chitin staining; red arrow refers to the old epidermis, white arrow refers to the new epidermis; blue signal indicates chitin. (**C**): Scanning electron microscopy of the epidermal surface. (**D**): The expression levels of Chitin synthetase and chitinase (*** *p* < 0.001, **** *p* < 0.0001.).

To observe whether changes occurred in the epidermal tissue, we dissected the control and *BmMBNL*-KO individuals during the molting period of the fourth-instar larvae and processed the dissected epidermis with paraffin wax sectioning. H&E staining and chitin staining were performed on the epidermal sections. The results showed that both *BmMBNL*-KO individuals and control individuals were at the stage of epidermal cell proliferation and new cuticle formation, but the old and new cuticles had separated in the control group. In contrast, the old cuticle of the knockout individuals did not separate from the new cuticle (Figure 4A). Figure 4B shows a reduction in chitin synthesis in the epidermis of *BmMBNL*-KO individuals. Scanning electron microscopy (SEM) results indicated that the absence of *BmMBNL* had no effect on the surface structure of the silkworm epidermis (Figure 4C). To determine the cause of reduced chitin synthesis in *BmMBNL*-KO individuals, we extracted RNA from the epidermis of *BmMBNL*-KO individuals during the fourth molting stage and reverse-transcribed it into cDNA. We then measured the expression levels of chitin synthase A, as well as chitinase 5 and chitinase 10, in the epidermis. The results indicated that the expression of these three genes was significantly lower in *BmMBNL*-KO individuals (Figure 4D).

### 3.4. BmMBNL Regulates Silkworm Molting by Influencing the Composition of the Epidermis and the Expression of Chitinase

**Figure 5:**
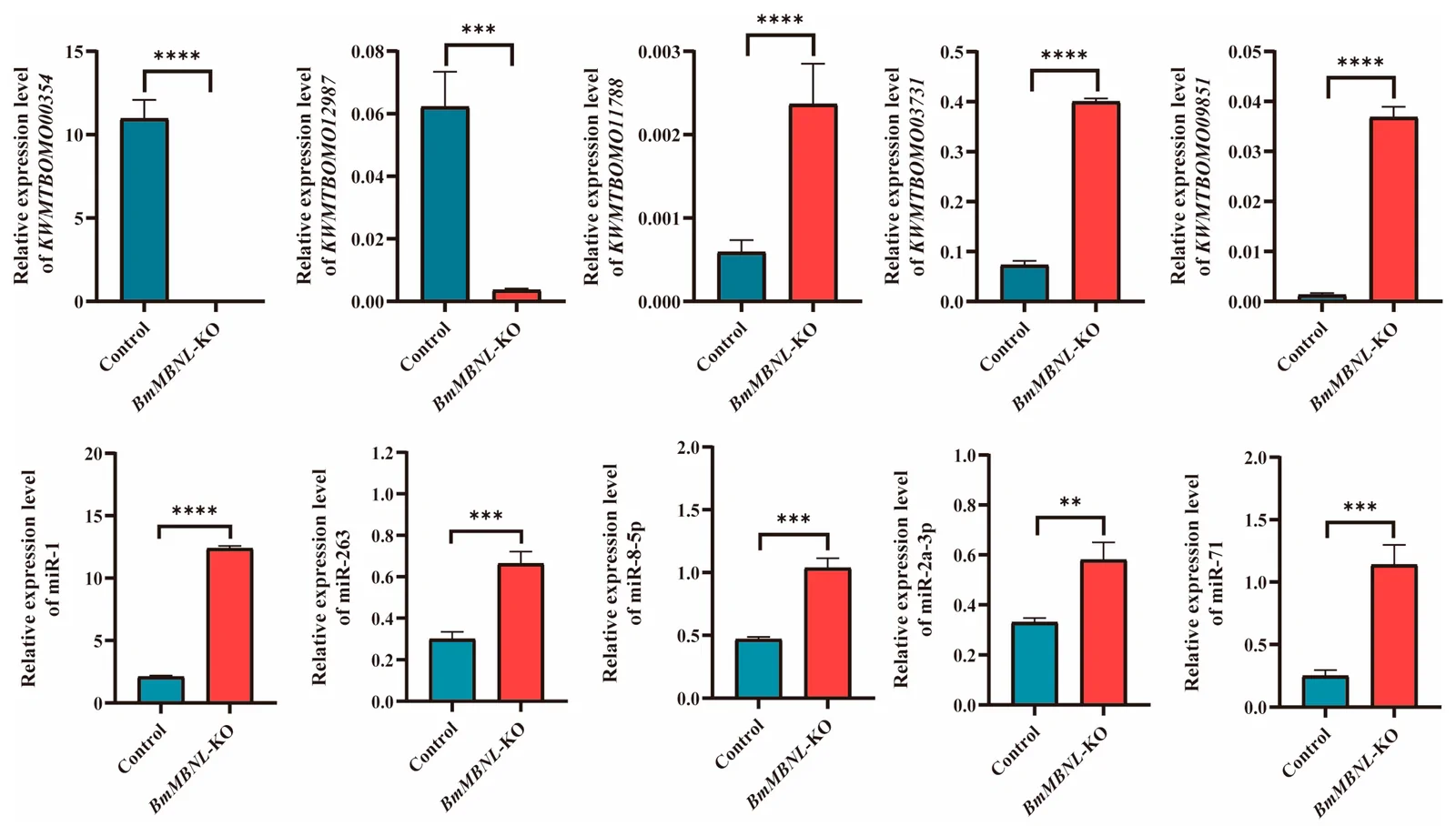
Identification and RT-qPCR validation of differentially expressed miRNAs and genes in *BmMBNL*-KO silkworms (** *p* < 0.01, *** *p* < 0.001, **** *p* < 0.0001.).

**Figure 6:**
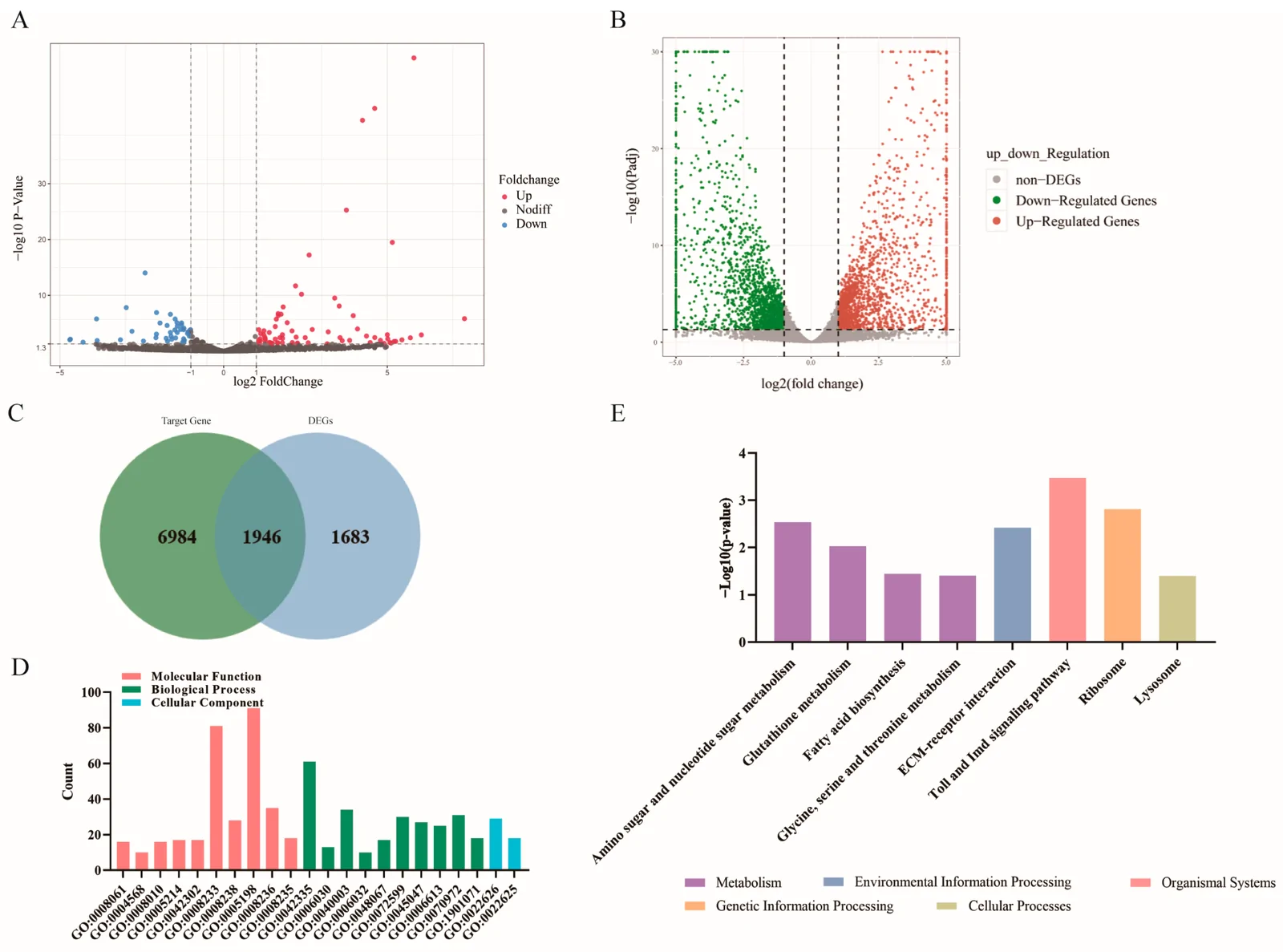
miRNA sequencing and RNA sequencing data analysis of *BmMBNL*-KO epidermis. (**A**): Volcano diagram of differentially expressed miRNAs. Dashed lines mark the screening thresholds: |log_2_(fold change)| > 1 and adjusted *p* < 0.05. (**B**): Volcano diagram of DEGs. Dashed lines mark the screening thresholds: |log_2_(fold change)| > 1 and adjusted *p* < 0.05. (**C**): A shared gene count between targets of differential miRNAs and DEGs. (**D**): GO functional enrichment of genes that are both differential miRNA targets and DEGs. (**E**): KEGG signaling pathway enrichment of genes that are both differential miRNA targets and DEGs.

To further elucidate the mechanism by which *BmMBNL* knockout affects silkworm molting, miRNA-seq and RNA-seq analyses were conducted on the epidermis of control and *BmMBNL*-KO individuals at the fourth molting stage, with three replicates per sample. Selected differentially expressed genes and miRNAs from sequencing were verified via RT-qPCR, showing consistent expression trends with the sequencing results (Figure 5). The results revealed a total of 114 differentially expressed miRNAs affected by the knockout, including 71 upregulated and 49 downregulated miRNAs (Figure 6A). Additionally, there were 3629 significantly differentially expressed genes, comprising 1785 upregulated and 1844 downregulated genes (Figure 6B). The target genes of the differentially expressed miRNAs were predicted, yielding a total of 8930 genes, of which 1946 were differentially expressed. GO functional enrichment analysis was performed on these genes, indicating that the biological processes and molecular functions were the primary enriched categories. The specifically enriched GO terms mainly included those related to epidermal development, such as chitinase binding, chitinase activity, epidermal structure, epidermal development, chitin metabolism, chitin degradation, and epidermal pigmentation. Most of the genes enriched in these terms were chitin synthetase, chitinase, and epidermal proteins, and their expression generally showed a downward trend.

The KEGG signaling pathway enrichment analysis identified four metabolic pathways, namely amino sugar and nucleotide sugar metabolism, glutathione metabolism, fatty acid biosynthesis, and glycine, serine, and threonine metabolism. Notably, the genes enriched in the amino sugar and nucleotide sugar metabolism pathway were associated with the synthesis and degradation of chitin. These include the enzymes necessary for the chitin synthesis pathway, such as chitin synthetase and chitinase, which predominantly exhibit a downregulated trend. These genes are regulated by 14 differentially expressed miRNAs (Table 2), with the majority of these miRNAs showing upregulated expression levels.

## 4. Discussion

The insect epidermis is composed of three complex layers: the envelope, epicuticle, and procuticle [29]. Chitin is a key component of the insect cuticle, accounting for 20% to 50% of the dry weight of the insect body. Within the epidermal tissue, chitin synthase A polymerizes UDP-N-acetylglucosamine to form chitin. The chitin-binding protein Knk is involved in the formation of the chitin lamellar structure of the insect cuticle and prevents the degradation of the new cuticle by chitinases in the molting fluid [30]. In the red flour beetle, Tribolium castaneum, Retroactive (Rtv) plays a role in the transport of Knk protein. Interference with Rtv leads to the accumulation of KnK in epidermal cells, which cannot be transported to the procuticle and thus fails to protect the new cuticle [31]. Chitin and cuticular proteins are the main parts of the cuticular structure; therefore, the degradation of the old cuticle involves two processes: the degradation of chitin and the degradation of cuticular proteins. The old cuticle of insects is a tight structure of chitin and cuticular proteins, with cuticular protein degradation occurring prior to chitin degradation [32]. The molting fluid contains many proteolytic substances, including cathepsins, protease inhibitors, and chitinases, all of which are involved in the synthesis of the new cuticle and the degradation of the old one. Proteases in the molting fluid can degrade cuticular proteins in the old cuticle, thereby exposing chitin. Chitin synthases and chitinases play important functions in insect molting. In Tribolium castaneum, interference with TcCHT5 during the pupal stage prevents the beetle from molting from pupa to adult, leading to death [33]. Interference with CHT5 in locusts also leads to nymphal molting defects, with the nymphs’ bodies trapped in the old cuticle, leading to death because the old cuticle encases the appendages and prevents the adults from moving freely [34]. It has been found that RNAi-mediated HcCht5 silencing prevents molting and leads to larval death, depending on the timing of dsRNA injection, and HcCht5 silencing down-regulates genes associated with chitin metabolism, ecdysteroid signaling pathway, and detoxification metabolism [34]. After RNAi with PxCHT5, PxCHT7, and PxCHT10, molting defects occurred [35]. In general, chitinase genes play an important role in insect molting, and their reduced levels can lead to insect molting defects. In this study, we used CRISPR/Cas9 technology to systematically knock out *BmMBNL* in silkworms, which led to a significant reduction in chitinase levels in *BmMBNL*-KO, resulting in molting defects.

MBNL is a class of ancient metazoan RNA-binding proteins characterized by the presence of one or two evolutionarily conserved tandem CCCH-type zinc finger motifs [36]. These motifs enable the recognition of the 5′-YGCY-3′ binding motifs in target RNA [37]. In addition to splicing regulation, MBNL also influences gene expression through miRNA processing [14,38,39]. MiRNA plays an important regulatory role in the growth and development of insects, especially during periods of molting or metamorphosis. In locusts, miR-71 and miR-263 can regulate the expression of Chitin Synthetase 1 (CHS1) and Chitinase 10 (CHT10), affecting the synthesis and degradation of chitin in the epidermis, which leads to molting defects and death [40]. Downregulation of miR-184 leads to molting failure in locusts [40]. In the brown planthopper, miR-8-5p and miR-2a-3p respond to 20-hydroxyecdysone (20E) and target membrane-bound trehalose phosphatase (Tre-2) and N-acetylglucosamine mutase (PAGM), leading to a decrease in epidermal chitin content and molting disorder with reduced survival rates upon overexpression of miR-8-5p and miR-2a-3p [41]. In this study, we found that *BmMBNL* knockout significantly affected enzyme genes involved in the chitin synthesis and degradation pathway, and miRNA levels regulating these genes were also changed. Therefore, we speculate that the molting defect caused by *BmMBNL* deletion may be mediated via miRNA regulation, which potentially influences chitin synthesis and degradation in the silkworm epidermis, resulting in reduced chitin synthesis and failure of chitin degradation in the old epidermis, thereby leading to molting difficulty. It should be noted that many downstream molecular events described above are consistent with established knowledge: the essential roles of chitinases in molting and the ability of miRNAs to modulate insect chitin metabolism have been widely reported. Accordingly, the novelty of the present study does not reside in these downstream pathways. Instead, the main contribution lies in identification and first functional characterization of the upstream regulator *BmMBNL* in silkworm molting.

Molting is a crucial process in the growth and development of silkworms, allowing them to shed their old cuticle and make room for further growth and development. However, if the genes involved in molting are disrupted, the silkworms will be unable to complete the molting process and will die. Therefore, in-depth research on the mechanisms of insect molting gene regulation will help us understand insect growth and development and provide researchers with potential pesticide targets. This study suggests that the *BmMBNL* gene participates in the regulation of epidermal development as well as chitin synthesis and degradation, and may contribute to molting control in silkworms. If the specific binding sites of the *BmMBNL* protein can be identified, they could serve as candidate targets for pesticide design. However, further research is needed to fully elucidate how *BmMBNL* is involved in epidermal development and chitin synthesis and degradation. In addition, the knockout of *BmMBNL* affects the regulation of epidermal development by miRNAs, and the specific target genes need to be further verified.

This study has two main limitations. First, only representative histological images are provided, without quantitative analysis of cuticle thickness and chitin fluorescence. Second, the current results demonstrate correlative changes rather than causal relationships. Direct *BmMBNL*–miRNA interaction, miRNA-target validation, rescue experiments and epistasis analysis are absent. The proposed regulatory model is a working hypothesis, and further experimental verification will be carried out in follow-up work.

## 5. Conclusions

In summary, this study suggests that *BmMBNL* plays an essential role in the molting process of the silkworm *Bombyx mori*. *BmMBNL* exhibits spatiotemporal expression patterns, with enrichment in the epidermis and upregulation during molting stages. Knockout of *BmMBNL* leads to molting defects and thinning of the chitin layer between the old and new epidermises. Mechanistically, *BmMBNL* deficiency upregulates a set of microRNAs (miR-1, miR-71, miR-263, miR-8-5p, and miR-2a-3p), which in turn suppresses chitinase expression, ultimately impairing successful molting. These findings provide novel insights into the miRNA-mediated regulatory network underlying insect molting and imply that *BmMBNL* may serve as a molecular link between miRNA processing and cuticle remodeling. Targeting this pathway may offer new strategies for pest control by disrupting the molting process in lepidopteran insects.

## Figures and Tables

**Table 1:** Sequence summary of primers.

Name	Sequence
>*BmMBNL*-qPCR-F	5′-TGCTGAACGGCAAGGACTC-3′
>*BmMBNL*-qPCR-R	5′-TGTTGTGGCGGATGGAAGT-3′
>*BmChsA*-F	5′-AGAAACGAAAGGATGGGACG-3′
>*BmChsA*-R	5′-GAAGGTGACCAGGTAAGCGAC-3′
>*BmCHT5*-F	5′-CTGAAGGCGGCTCCAAGTA-3′
>*BmCHT5*-R	5′-TCCCAGTCCAGATCCAAACC-3′
>*BmCHT10*-F	5′-ACCGCTTTATGGTCGCAGTT-3′
>*BmCHT10*-R	5′-CGTTGATGGGGTATCGTATGTC-3′
>*SW22934*-F	5′-TTCGTACTGCTCTTCTCG-3′
>*SW22934*-R	5′-CAAAGTTGATAGCAATTCCCT-3′

**Table 2:** Differentially expressed miRNAs involved in amino sugar and nucleic acid sugar metabolism in *BmMBNL*-KO knockout individuals. Up, significantly upregulated; Down, significantly downregulated.

miRNA Name	Up/Down-Regulation
bmo-miR-274-5p	Up
bmo-miR-34-5p	Up
bmo-miR-1175-3p	Up
bmo-miR-3388-3p	Up
bmo-miR-2843-1-3p	Down
bmo-miR-6497-3p	Up
bmo-miR-3000	Up
bmo-miR-6497-5p	Up
bmo-miR-9b-3p	Up
bmo-miR-2738	Up
novel-m0026-5p	Down
novel-m0539-5p	Up
novel-m0310-5p	Down
novel-m1169-3p	Down

## Data Availability

The original contributions presented in this study are included in the article. Further inquiries can be directed to the corresponding author.
